# NeTFactor, a framework for identifying transcriptional regulators of gene expression-based biomarkers

**DOI:** 10.1038/s41598-019-49498-y

**Published:** 2019-09-10

**Authors:** Mehmet Eren Ahsen, Yoojin Chun, Alexander Grishin, Galina Grishina, Gustavo Stolovitzky, Gaurav Pandey, Supinda Bunyavanich

**Affiliations:** 1 Icahn Institute for Genomics and Multiscale Biology and Department of Genetics and Genomic Sciences, Icahn School of Medicine at Mount Sinai, New York, NY USA; 2 Division of Allergy & Immunology, Department of Pediatrics, Icahn School of Medicine at Mount Sinai, New York, NY USA; 3 IBM T.J. Watson Research Center, Yorktown Heights, New York, NY USA

**Keywords:** Gene regulatory networks, Gene expression

## Abstract

Biological and regulatory mechanisms underlying many multi-gene expression-based disease biomarkers are often not readily evident. We describe an innovative framework, *NeTFactor*, that combines network analyses with gene expression data to identify transcription factors (TFs) that significantly and maximally regulate such a biomarker. NeTFactor uses a computationally-inferred context-specific gene regulatory network and applies topological, statistical, and optimization methods to identify regulator TFs. Application of NeTFactor to a multi-gene expression-based asthma biomarker identified ETS translocation variant 4 (ETV4) and peroxisome proliferator-activated receptor gamma (PPARG) as the biomarker’s most significant TF regulators. siRNA-based knock down of these TFs in an airway epithelial cell line model demonstrated significant reduction of cytokine expression relevant to asthma, validating NeTFactor’s top-scoring findings. While PPARG has been associated with airway inflammation, ETV4 has not yet been implicated in asthma, thus indicating the possibility of novel, disease-relevant discovery by NeTFactor. We also show that NeTFactor’s results are robust when the gene regulatory network and biomarker are derived from independent data. Additionally, our application of NeTFactor to a different disease biomarker identified TF regulators of interest. These results illustrate that the application of NeTFactor to multi-gene expression-based biomarkers could yield valuable insights into regulatory mechanisms and biological processes underlying disease.

## Introduction

Biological and regulatory mechanisms underlying most multi-gene expression-based disease biomarkers are often not readily evident. Using RNA sequencing (RNAseq)^1^ and machine learning^2^ in a well-characterized cohort of asthmatics and controls, we recently identified a nasal brush-based biomarker of asthma^3^. This biomarker consists of 90 genes, whose expression is interpreted through a logistic regression function^3^. Although our nasal biomarker of asthma produced accurate (AUC 0.994) and specific classification of asthma^3^, the biological and regulatory mechanisms underlying its performance were not readily evident. For instance, although the genes in the biomarker had a higher tendency to be differentially expressed (Kolmogorov-Smirnov statistic = 0.289, FDR = 2.73 × 10^−37^), only non-specific pathways such as defense response (fold change = 2.86, FDR = 0.006) were enriched in these genes, and only a minority have been previously studied in the context of asthma^3^. A gene expression-based biomarker like ours is expected to include genes known to associate with the target disease. However, it is also possible and even likely, given our incomplete understanding of complex diseases such as asthma, that genes not previously associated with the disease can provide information that is useful to the classification, and perhaps to the disease process itself. Indeed, such an approach has led to important results in other disease areas such as cancer^4–7^, illustrating the idea that RNA traits can serve as sensitive sensors of one state (e.g. disease) relative to another (e.g. healthy) beyond known associations with established disease-related pathways. It is intriguing to consider what further dissection of our asthma and other biomarkers could yield as insights into biologic mechanisms relevant to asthma and other diseases.

Here we describe a novel framework that combines network analyses with RNA sequence (RNAseq) data to identify transcription factors (TFs) significantly regulating a disease biomarker. This framework, named *NeTFactor* (Network-identified Transcription Factor), uses a computationally inferred context-specific gene regulatory network (GRN)^8^ to guide the analysis. Such a GRN consists of directed edges denoting interactions between regulators (e.g. TFs) and their target(s) (e.g. gene(s) they regulate). NeTFactor utilizes the structure and constituents of such a GRN to identify the regulators, specifically TFs, that most significantly regulate the genes underlying the biomarker. To illustrate the utility of our framework, we applied NeTFactor to identify the most significant TF regulators of our nasal gene expression-based asthma biomarker^3^ and then experimentally validated the identified regulators using silencing RNA (siRNA)^9^ in airway epithelial cell line models. Further, we show that that NeTFactor’s results are robust when the gene regulatory network and biomarker are derived from independent data and additionally demonstrate application of NeTFactor to a different disease biomarker.

Biomolecular networks, including GRNs, have been widely used to glean useful insights into biological processes and how the dysregulation of the constituent interactions may lead to disease^8,10–12^. In particular, network analyses have been used to identify disease-related genes and regulators, often connected through interactions in the network, representing a subnetwork or module^13–15^. Master Regulator Analysis (MRA)^16^ and its variants^17^ represent such an approach where a GRN is used to directly identify TF regulators that are expected to be associated with the target disease or phenotype. In parallel, similar to our asthma biomarker, multi-gene expression-based biomarkers have been developed in other disease areas, e.g., breast cancer prognosis^4,18^. The goal of this study was to analyze a GRN to identify the most significant set of key TF regulators of the set of genes constituting a separately identified biomarker, namely our asthma biomarker. This is complementary to investigating the constituent genes of the biomarker individually, as well as only identifying TF regulators associated with the target disease or phenotype using methods like MRA. In other words, we used computational and systems biology principles^19–21^ to develop a novel framework that integrates machine learning- and network-based analyses of complex biomolecular data.

## Results

**Figure 1:**
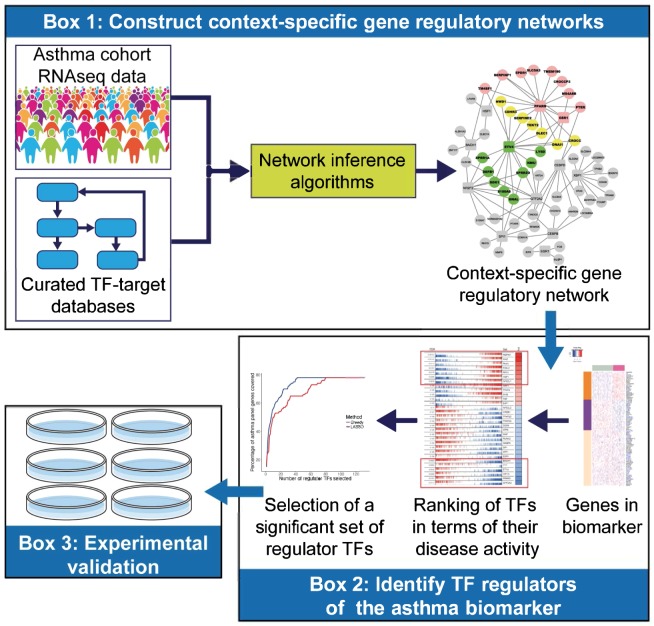
Study flow for the identification and validation of transcription factor (TF) regulators of a gene expression-based biomarker of asthma^3^ using the proposed NeTFactor framework. Box 1 denotes the first step of NeTFactor, namely the inference of gene regulatory networks (GRNs) from the datasets that yielded the original biomarker. Box 2 represents steps 2–4 of NeTFactor which identify the most significant set of likely TF regulators, which are themselves active in the disease and regulate a significant fraction of genes constituting the biomarker. Box 3 depicts siRNA-mediated knock-down experiments in an airway epithelial cell line model employed to experimentally validate the identified regulators.

Our study comprised multiple steps (Fig. 1), including the application of NeTFactor to construct a context-specific gene regulatory network (Box 1) and identify TF regulators of the biomarker (Box 2), followed by experimental validation of the inferred TF regulators (Box 3).

### Development of NeTFactor and its application to nasal RNAseq data and the asthma biomarker

#### Generation of a context-specific gene regulatory network (GRN)

**Figure 2:**
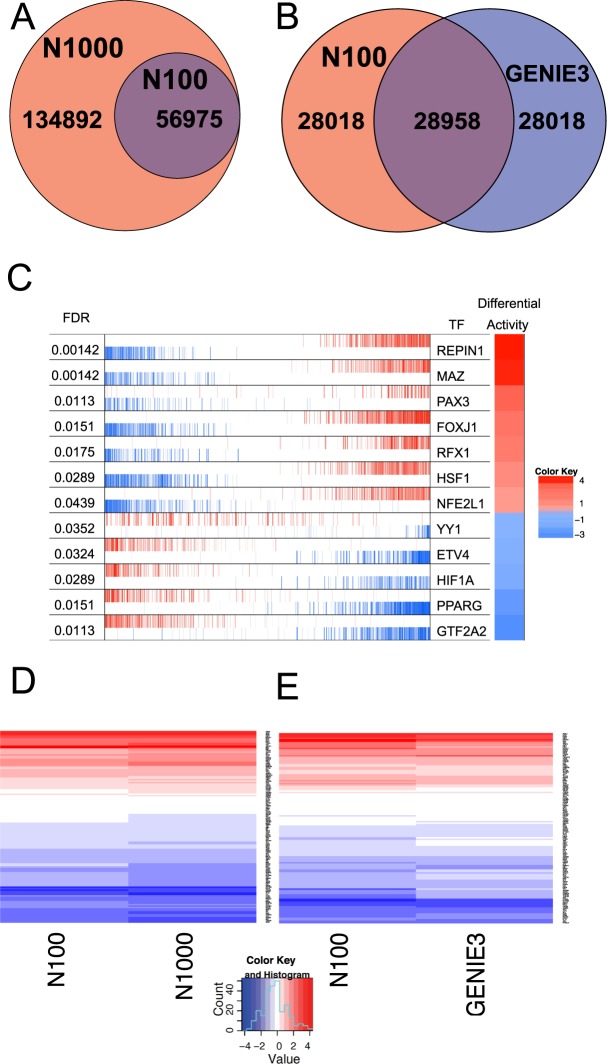
Derivation of context-specific gene regulatory networks (GRNs) from and application of the VIPER algorithm^30^ to a nasal RNAseq data set. (**A**) Venn diagram showing the overlap between the TF→target gene interactions constituting GRNs generated by applying the ARACNE algorithm^23^ to a nasal RNAseq dataset using 100 (N100) and 1000 (N1000) bootstraps shows that the former is completely contained in the latter. (**B**) Venn diagram showing the statistically significant overlap of the TF→target gene interactions constituting the N100 network and the GRN of the same size inferred using the GENIE3 algorithm^29^ from the same dataset (Fisher’s exact test p < 2.2e-16). (**C**) Transcriptional activities of the 12 transcription factors most significantly differentially active in asthma. The first column indicates VIPER FDR value for differential activity (FDR ≤ 0.05) of TFs active in asthma. All genes in the nasal RNAseq data are graphically summarized in the second column, where each vertical line represents a gene, and the genes are rank-sorted left to right from most down-regulated to most up-regulated in asthma vs normal subjects. Blue and red bars indicate negative and positive regulation, respectively, of each gene by the TF shown in the third column. The fourth column shows VIPER-inferred differential activity of TFs, with red and blue entries indicating more and less activity of the TF in asthma, respectively. (**D**) Heatmap of VIPER-inferred activity scores of TFs constituting the N100 and N1000 networks. The scores are very highly correlated (Pearson’s correlation coefficient = 0.987, p < 2.2e-16). (**E**) Heatmap of VIPER-inferred activities of TFs constituting the N100 network and the GRN of the same size inferred using the GENIE3 algorithm. The scores are very highly correlated (Pearson’s correlation coefficient = 0.969, p < 2.2e-16).

The first step of NeTFactor is the derivation of a base GRN that reflects the biological context, such as the same tissue of origin, of the target biomarker. For this, in our study, the application of the ARACNE algorithm^22–24^ to nasal RNAseq data from a case-control asthma cohort (n = 150) (Supplementary Table 1) yielded a base GRN consisting of 56976 interactions between 132 TFs and 11049 genes. Since this network was inferred from *nasal* gene expression data, it is expected to be directly relevant to our *nasal* brush-based asthma biomarker as well as to asthma overall, given shared biology between the nasal and bronchial airways^3,25,26^. Applying ARACNE with 1000 bootstraps instead of the default value of 100 generated a much larger but fully encompassing GRN (Fig. 2A), indicating that the core network was preserved between these variations of the algorithm. Although there were no set criteria for selecting the size of the final GRN, we observed that the base network was the closest in size to the total number (66883) of curated TF → target gene interactions in MSigDB^27,28^ version 5.1, which was also the source of TFs used to derive the ARACNE networks. To capture the extent of our current knowledge of GRNs, we used the 100 bootstrap base GRN for further analyses. However, due to the general lack of knowledge about human TFs and their putative target genes, this network only included 78 of the 90 (87%) genes in the asthma biomarker, placing an upper limit on how many of these genes could be regulated by the TFs in the GRN.

We also generated a GRN containing the same number of edges as the base ARACNE network using the GENIE3 algorithm^29^, and found that the two networks overlapped significantly (Fig. 2B; Fisher’s exact test p < 2.2e-16). This supported the robustness of using ARACNE within NeTFactor and its resulting GRN.

#### VIPER identifies 12 asthma-associated TFs in the GRN

NeTFactor next examines if a TF is differentially active in the disease under consideration, such as asthma in our application study, for the TF to be considered a regulator of the target biomarker under consideration. To identify such TFs, we applied the VIPER algorithm^30^ to the base GRN and the RNAseq data to identify TFs that are differentially active between asthmatic and control subjects. The output of VIPER consisted of a normalized enrichment score (NES), which was positive for TFs that were more active in asthma and negative for TFs more active in control subjects, as well as the associated false discovery rate (FDR_VIPER_) value for each NES. Figure 2C shows the 12 TFs found to be differentially active in asthma (FDR_VIPER_ ≤ 0.05). These TFs included HSF1, which has been reported to affect airway hyperresponsiveness and airway inflammation in mice with asthma^31^.

We also tested the sensitivity of the VIPER results to the choice of the input GRN and the algorithm used to infer it. For this, we compared the TF activity scores (NESs) inferred from the base GRN with those inferred from the ARACNE 1000 bootstrap and GENIE3 networks. As shown in Fig. 2D (Pearson’s correlation coefficient = 0.99, p < 2.2e-16) and 2E (Pearson’s correlation coefficient = 0.97, p < 2.2e-16), the scores from these alternative GRNs were highly correlated with those from the base GRN. These results strongly support that the TF activity scores and downstream analyses utilizing them would be robust to the choice of the network inference algorithm and the resulting GRN.

#### Context-specific regulators of the asthma biomarker

The next step of NeTFactor assesses if a TF significantly regulates the genes constituting the target biomarker. For this, we calculated the likelihood that a particular TF regulated our asthma biomarker by conducting a Fisher’s exact test^32^ for the statistical significance of the overlap between the set of genes regulated by each TF in the base nasal GRN (i.e. its *regulon*) and the member genes of the asthma biomarker. This was followed by correction for multiple hypothesis testing using the Benjamini-Hochberg procedure^33^, which yielded a regulation likelihood for each TF (FDR_BIOMARKER_) that was used in subsequent analyses (Supplementary Table 2). The most significant of these regulators (FDR_BIOMARKER_ ≤ 0.05) included XBP1, which modulates endoplasmic reticulum stress in type 2 airway inflammation^34^ and mucin production^35^ that may relate to how the well-replicated asthma locus ORMDL affects asthma^36^.

#### Convex optimization identifies the most significant set of asthma-active TFs that most significantly and non-redundantly regulate the asthma biomarker

The previous two steps of NeTFactor identified TFs that were disease-active and likely to regulate the target biomarker. The final step of the framework aims to identify *the most significant* set of TFs that scores highly on both these aspects, but has as little redundancy as possible among the sets of biomarker genes they regulate, thus maximizing the coverage of the biomarker. We considered several approaches for determining this *most significant* set. The *greedy* approach incrementally selects TFs ranked by the number of biomarker genes they target in the GRN, not taking the redundancy among target biomarker gene sets into account. In contrast, the *LASSO-*based convex optimization approach calculates a global weight for the TFs that incorporates the non-redundancy of their target biomarker gene sets, in addition to the FDR_VIPER_ and FDR_BIOMARKER_ likelihoods calculated above. The LASSO approach covers a higher total number of biomarker genes with fewer TFs than the greedy approach due to LASSO’s better control of redundancy (Supplementary Fig. 1). For instance, with 40 selected TFs, the LASSO approach covered approximately 15 more biomarker genes than the greedy approach.

**Figure 3:**
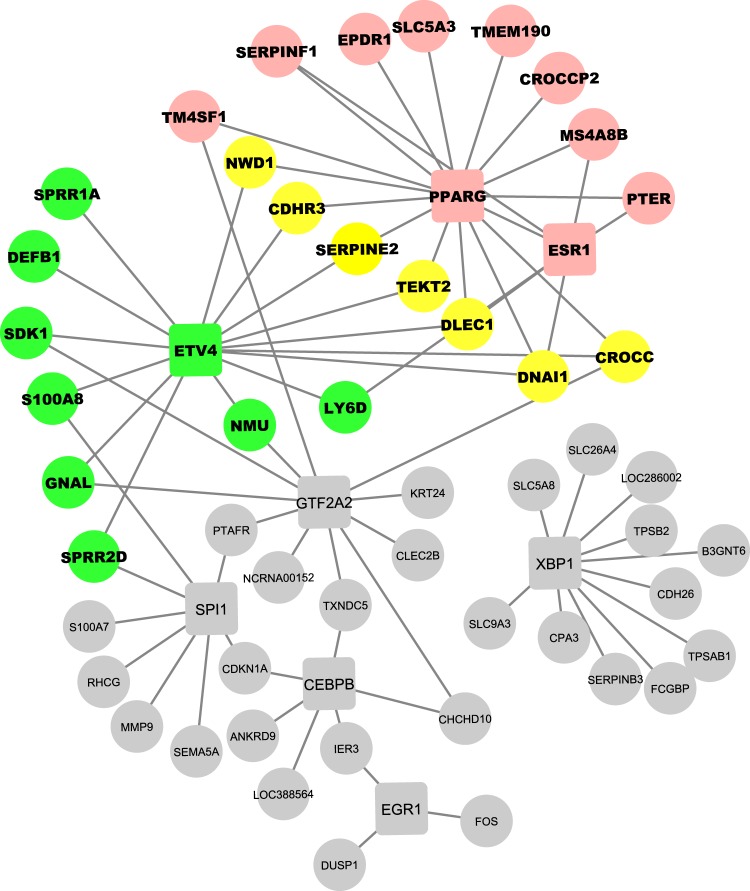
Subnetwork of the base nasal GRN consisting of the seven regulator TFs listed in Table 1, and their target asthma biomarker genes, denoted by squares and small filled circles respectively. The most significant regulators of the biomarker, ETV4 and PPARG, and the genes regulated exclusively by each of them are shown in green and pink respectively, and those regulated by both TFs are shown in yellow. Other TFs and target genes are shown in grey, with the exception of ESR1, which is itself regulated by PPARG. Figure made using CytoScape^71^.

Based on these observations, we examined the results of the LASSO approach in greater detail, which revealed that there was substantial variation in the weights of the TFs, especially due to differences in FDR_VIPER_ and FDR_BIOMARKER_. Details of the top seven weighted TFs and all 132 TFs in the base nasal GRN are provided in Table 1 and Supplementary Table 2 respectively. In particular, ETV4 and PPARG were the only TFs that met three criteria: (1) highly weighted, (2) significantly differentially active in asthma (FDR_VIPER_ ≤ 0.05), and (3) significantly regulating the asthma biomarker (FDR_BIOMARKER_ ≤ 0.05) (Table 1). Just these two TFs regulated 24 (27%) of the asthma biomarker genes, including SERPINE2^37^ and CDHR3^38^, asthma-associated genes co-regulated by ETV4 and PPARG. Gene Ontology enrichment analysis, conducted using MSigDB)^27,28^, of the GRN subnetwork regulated by ETV4 and PPARG (Fig. 3) included terms highly relevant to disease processes in asthma, including *response to corticosteroid* (FDR = 2.94 × 10^−5^), *regulation of immune system process* (FDR = 8.92 × 10^−4^), and *innate immune response* (FDR = 5.89 × 10^−3^). Given these results, we focused on ETV4 and PPARG in our experimental validation efforts.

**Table 1:** The top seven TFs (first column) ranked by LASSO weights (second column) produced by the final step of NeTFactor, indicating the TF’s likelihood of regulating the 90-gene asthma biomarker as significantly and non-redundantly as possible.

TF	LASSO weight	FDR_VIPER_	FDR_BIOMARKER_	Number of biomarker genes regulated	Cumulative number of biomarker genes regulated
*PPARG*	*1*.*072*	*0*.*0151*	*0*.*006*	16	16
*ETV4*	*1*.*0*15	*0*.*03*24	*0*.*00007*	*15*	*24*
GTF2A2	1.014	0.0113	0.395	11	30
EGR1	1.006	0.566	0.286	3	33
SPI1	1.003	0.61	0.363	8	38
CEBPB	1.0008	0.887	0.062	7	41
XBP1	1.0005	0.998	0.0002	11	52

The FDR values calculated in the two preceding steps of NeTFactor are also shown for reference, along with the number of biomarker genes regulated by each TF, as well as genes cumulatively regulated by it and all the TFs preceding it. These results show that ETV4 and PPARG are the strongest TF regulators of the asthma biomarker, as they are the only ones that are significantly associated with asthma (FDR_VIPER_ ≤ 0.05) as well as significant regulators of the biomarker (FDR_BIOMARKER_ ≤ 0.05).

### Experimental validation of NeTFactor findings

To test the results from NeTFactor, we chose to investigate how knockdown of the regulatory TFs prioritized by NeTFactor (ETV4 and PPARG) in nasal epithelial cell line models would affect the production of inflammatory cytokines involved in asthma^39–42^. We chose to employ a nasal epithelial cell line to optimize context-specific validation, given the asthma biomarker is based on nasal gene expression, and we also used nasal RNAseq data as input for NeTFactor. Recognizing the limitations of cell lines as a model for *in vivo* inflammation, we first conducted a series of pilot experiments to assess the baseline responsiveness of a commercially available nasal epithelial cell line (HNEpC) to inflammatory stimulation. In response to the immunostimulant polyinosinic:polycytidylic acid (poly(I:C)), this cell line produced detectable amounts of IL6, IL8, TGFβ, CCL2, TSLP, and CCL17, with the highest levels noted for IL6 and IL8 (Supplementary Table 3). There was no detectable production of IL25 or IL33, and the cell line was less responsive to stimulation with lipopolysaccharide (LPS) or cytosine–phosphate–guanosine (CpG). With these pilot results as background, we designed our experimental validation of NeTFactor findings to include the measurement of IL8 and IL6 following stimulation with poly(I:C) in the nasal epithelial cell line model with and without siRNA knockdown of ETV and PPARG.

**Figure 4:**
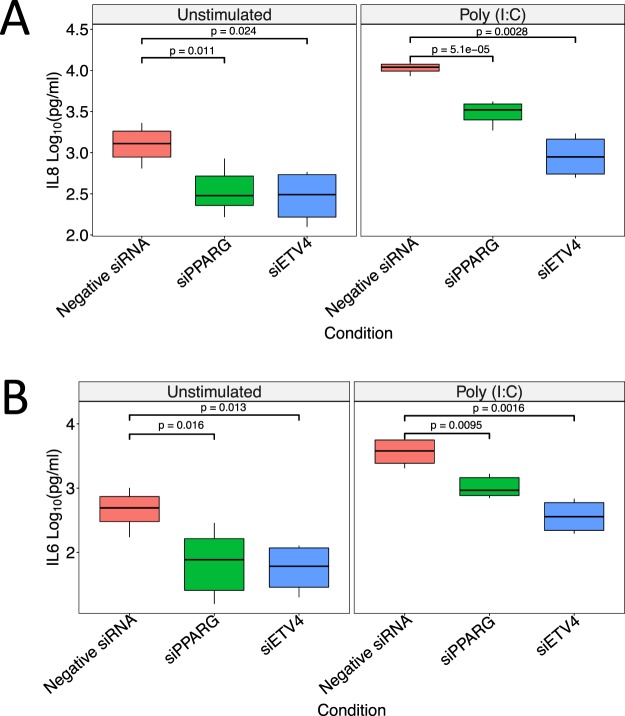
Experimental Validation of NeTFactor Predictions. Nasal epithelial cell lines with siRNA-mediated knockdowns of PPARG (siPPARG) and ETV4 mRNA (siETV), as well as negative siRNA control (Negative siRNA), were stimulated with Poly (I:C) and also left unstimulated. The concentrations of IL8 (Panel A) and IL6 (Panel B) at 24 hours are shown.

Figure 4 shows that both at baseline and in response to inflammatory stimulation with poly(I:C), the nasal epithelial cell line with ETV4 knocked down by siRNA (siETV4+) produced significantly smaller quantities of IL8 (Fig. 4A) and IL6 (Fig. 4B) compared to the negative siRNA control with intact ETV4. Similarly, the nasal epithelial cell line with PPARG knocked down by siRNA (siPPARG) yielded significantly lower IL8 (Fig. 4A) and IL6 (Fig. 4B) compared to negative siRNA control. These findings supported our expectation that levels of IL8 and IL6 would be repressed both at baseline and more significantly with knock down of ETV4 and PPARG based on NeTFactor’s results and our understanding of these cytokines in inflammation.

### NeTFactor’s performance when the biomarker and GRN are derived from different gene expression datasets

Up to this point, we assessed NeTFactor’s performance when the GRN in its first step was derived from the same gene expression dataset from which the target biomarker was also identified. To assess NeTFactor’s performance when the GRN and biomarker are derived from different datasets, we derived a GRN from an independent nasal gene expression dataset from a cohort of asthmatic children and controls^43^ different from the primary dataset from which the 90-gene biomarker was identified. Application of NeTFactor’s steps to the GRN derived from the independent cohort yielded a ranked list of TF regulators that largely overlapped with the ranked list obtained from when the GRN and biomarker were derived from the same dataset (Supplementary Table 4). Specifically, the top 10 ranked TFs from the two applications of NeTFactor significantly overlapped (Fisher’s exact test p = 0.0019), indicating consistency between the top-ranked regulators identified by the framework when distinct GRNs are used. The top two ranked regulators from the primary application of NeTFactor, ETV4 and PPARG, were the second and third ranked regulators when NeTFactor was applied to the independently-derived GRN. Due to clinical differences between the primary^3^ and additional independent cohort^43^ used to derive the two GRNs, most prominently differences in age given the original cohort consisted only of adult subjects while the independent cohort included only children, we expectedly did not find the exact same ranking of regulators, although as a group, the top ranked regulators were significantly consistent.

### Application of NeTFactor to a different disease biomarker

To assess NeTFactor’s ability to generalize to biomarkers of other diseases and/or phenotypes, we applied NeTFactor to identify TF regulators of a biomarker of peanut allergic reactions^44^. In this study, peanut allergic children underwent double-blind, placebo-controlled oral challenges to peanut where peripheral blood samples for whole blood transcriptome profiling were obtained during each challenge (i.e. peanut challenge and placebo challenge)^44^. All of the children reacted to peanut and none reacted to placebo^44^. In this scenario, peanut allergic reaction was the target phenotype, and the two classes were reaction (i.e. which occurred when peanut was given during peanut challenge), and no reaction (i.e. no reaction when placebo was given)^44^. In the primary study of this cohort^44^, a series of analyses on the subjects’ whole blood transcriptome profiles were performed that identified 26 key driver genes of peanut allergic reaction (Supplementary Table 4 of ref.^44^). These 26 key driver genes were considered the target biomarker for NeTFactor.

Table 2 shows the top-ranked TF regulators of the peanut allergy biomarker identified by NeTFactor (full ranking of TFs in Supplementary Table 5). Specifically, these TFs were identified using the LASSO procedure in NeTFactor’s last step and also satisfied the conditions of having both FDR_VIPER_ and FDR_BIOMARKER_ ≤ 0.05, supporting both their significant association with disease and biomarker relevance. These top-ranked regulators included STAT6, a TF known to have a central role in allergy through its modulation of Th2 cell differentiation, cell surface marker expression, and class-switching of immunoglobulins^45^, as well as, NFIL3, which is induced by STAT6 and regulates IgE production, an immunoglobulin central to allergy^46^. STAT3 plays a pivotal role in immune responses, regulating B cells and CD4+ and CD8+ T cells^47^, and whose dysregulation has been linked to aberrent IgE production and allergy^48^. SPI1 regulates follicular B cell development and germinal center responses^49^, and VDR is the receptor for vitamin D3, where studies have suggested that vitamin D is associated with allergy outcomes^50,51^.

**Table 2:** Top-ranked TFs identified by NeTFactor for the peanut allergy biomarker, ranked by LASSO weights.

TF	LASSO weight	FDR_VIPER_	FDR_BIOMARKER_
NFIL3	0.951	1.12 × 10^−22^	0.0084
SPI1	0.914	2.44 × 10^−8^	0.0103
STAT3	0.698	1.41 × 10^−5^	3.34 × 10^−5^
STAT6	0.573	0.0122	0.0055
VDR	0.515	0.004	0.0114

The TFs that are significantly associated with peanut allergy reaction (FDR_VIPER_ < 0.05) and are significant regulators of the biomarker (FDR_BIOMARKER_ < 0.05) are shown here.

## Discussion

With rapid advances in genomic technology, several multi-gene expression-based biomarkers have been identified for diseases like asthma^3^, breast cancer^52^, stroke^53^ and Alzheimer’s disease^54^. Although some of these biomarkers are already used in clinical practice, such as MammaPrint and Oncotype DX for breast cancer prognosis^4,18^, their biological interpretation beyond examination of their individual constituent genes or enriched Gene Ontology terms or pathway is not commonly undertaken. In this paper, we have proposed the NeTFactor framework, which is designed to identify the most significant set of transcription factors (TFs) most likely to regulate such a biomarker. This is complementary to investigating the constituent genes individually or only identifying regulators associated with the target disease or phenotype using methods like MRA^16^. Based on NeTFactor’s findings when applied to our nasal brush-based biomarker of asthma^3^, we knocked down the identified regulator TFs in a nasal epithelial cell line model, finding that cytokine output was appropriately repressed as expected given our understanding of the role of these cytokines in inflammation and asthma. We also demonstrated that NeTFactor can be used to identify TF regulators of a peanut allergy biomarker^44^. Our findings demonstrate that NeTFactor can be successfully applied to identify TF regulators of multi-gene expression-based biomarkers, yielding valuable insights into disease-relevant biological processes and allowing us to gain more from biomarkers beyond their main role as classifiers or predictors.

NeTFactor requires as input a GRN, preferably a reliable context-specific one, the inference of which generally requires a sizeable gene expression data set. Our results show that the GRN may be derived from the same or different disease-relevant data set from which the biomarker is derived. However, in applications where such data may not be available, one can still use the NeTFactor algorithm by providing a generic network, such as the set of TF → target gene interactions in MSigDB^27,28^, as input. However, this may result in reduced sensitivity of the results, as NeTFactor will not have access to the biological context expected from the GRN. Another requirement of NeTFactor is a set of TFs that are needed for inferring the GRN, which we obtained from MSigDB in this study. If this set is not reliable or comprehensive, the resulting GRN and downstream analyses, especially the assessment of the constituent TFs’ disease-relevant activity (FDR_VIPER_) and enrichment of the biomarker genes among their targets (FDR_BIOMARKER_), may be adversely affected.

The final step of NeTFactor adopts a novel LASSO-based convex optimization approach to determine the most significant set of regulator TFs that maximizes the coverage of the biomarker genes. This approach has the benefit of collectively optimizing the relevant factors, namely FDR_VIPER_, FDR_BIOMARKER_, and non-redundancy among the target biomarker genes, for determining the most significant TF regulators of the biomarker. However, in certain cases, the solution of such an optimization problem may result in local optima, and consequently potentially false discoveries. More generally, due to the computational nature of NeTFactor, the possibility of false positive and negative results from the framework cannot be ruled out. The reliability of the results can be partially addressed by experimentally validating the results, as was done in this study, but that necessitates resources for this validation. These potential issues also indicate the possibility of developing alternative regulator prioritization methods that may improve the results of the LASSO approach used in this work.

Application of NeTFactor to a cohort of asthmatics and controls indicated that PPARG and ETV4 were the most likely regulators of an asthma biomarker. PPARG has been reported in some contexts to exert anti-inflammatory effects through the regulation of signaling in immune cells, including monocytes/macrophages, platelets, lymphocytes, and dendritic cells (DCs), as well as in epithelial, endothelial, and smooth muscle cells^55–57^. However, its role in asthma is more controversial with heterogeneous findings. While activation of PPARG has been associated with anti-inflammatory effects on airway^58–63^, PPARG signaling has more recently been shown to be critical for IL33–driven Th2 effector function in type-2 allergic airway responses, suggesting a contrasting pro-inflammatory role^63^. Furthermore, upregulation of PPARG in lung-resident CD11b+ DCs enhances migration to draining lymph nodes and Th2 priming capacity^63^.

Distinct from PPARG, far less is known about ETV4 (ETS variant 4) in the context of asthma. ETV4 belongs to the PEA3 (polyomavirus enhancer activator 3) subfamily of a larger E26 transformation-specific gene family of transcription factors^63–66^. ETV4 promotes morphogenesis of epithelial organs including lung during embryogenesis and plays a role in cell proliferation, growth, migration and apoptosis^64^. Our findings indicate a novel role of this transcription factor in asthma.

Our application of NeTFactor to a biomarker of peanut allergy^44^ revealed top-ranking TF regulators with established roles in immune regulation and allergy^45–51^. NeTFactor’s results suggest that their specific roles in peanut allergy in particular may be worth further study.

To conclude, we propose NeTFactor, an innovative framework that combines gene expression data and network analyses to identify the most significant set of transcription factor regulators of a multi-gene expression-based biomarker. Such regulators can yield valuable insights into regulatory mechanisms and disease-relevant biological processes related to their biomarkers, extending their utility beyond being mainly used as classifiers or predictors.

## Materials and Methods

### Primary study population and RNAseq data

In the primary study, we applied NeTFactor to the same development set of RNAseq-derived nasal gene expression data from 150 subjects with and without asthma that was used to identify the asthma biomarker in our earlier study^3^. The baseline characteristics of this study population are shown in Supplementary Table 1. This is one of the largest publicly available datasets of nasal gene expression from a well-characterized asthma cohort. The use of this rich data set for complementary analyses underlying our biomarker and NeTFactor is expected to improve our ability to identify regulatory network-derived explanations for the strong performance of our biomarker.

### NeTFactor

Below, we describe the four main steps of NeTFactor, which finds the most significant set of TF regulators for a given biomarker, explained in terms of its main application to our asthma biomarker and the associated nasal RNAseq dataset mentioned above:

1. Reverse engineering a context-specific Gene Regulatory Network (GRN): We applied the well-established and publicly available ARACNE algorithm^22–24^ to the RNAseq dataset to infer a GRN specific to the nasal tissue. Briefly, ARACNE^22,24^ uses mutual information^67^, a symmetric information theoretic similarity measure that accounts for possible non-linear relationship(s) between two entities (here, genes). Given a $sample\times gene$ expression matrix, ARACNE calculates mutual information between all pairs of genes (columns of the matrix), representing an initial fully connected network. Next, augmented with a list of putative TFs provided, ARACNE uses the data processing inequality (DPI)^67^ to remove interactions whose constituent information is sufficiently captured by the rest of the interactions, which generates the resulting GRN consisting of TF → target gene interactions^24^. Specifically, for all the cliques consisting of three gene $\leftrightarrow \,$gene edges, where some of the genes represent the putative TFs, ARACNE removes the edge with the lowest value of mutual information in accordance with DPI. The only exceptions to this process are the cases where this removal will retain a non-TF ↔ non-TF edge, which is not considered a transcriptional interaction, and is removed instead. Repeating this process throughout the original mutual information matrix generates the resulting GRN consisting of TF → target gene interactions.
  The Adaptive Partitioning (AP) version of ARACNE^23^ improves its computational efficiency and robustness using multiple bootstraps of the gene expression data and averaging the resulting network to create the final GRN. We applied this version with the default number of bootstraps (100) in the publicly available implementation^23^ to create the nasal GRN for further analysis, but also tested other numbers of bootstraps (e.g., 1000) to test the GRN’s dependence on this parameter’s value. 221 putative TFs for running ARACNE were obtained from the Molecular Signature Database (MSigDB)^27,28^ version 5.1 (accessed June 3^rd^, 2016).
2. Inferring disease-related activity of TFs in the GRN: To enhance the functional relevance of candidate regulator TFs, NeTFactor requires such TFs to be differentially active under disease conditions. For this, we applied the VIPER algorithm^30^ to the nasal GRN, along with the asthma/no-asthma status of the expression profiles used to derive the GRN. Specifically, VIPER was run with the sample-based permutation option to build the null hypothesis. The output of VIPER consists of a normalized enrichment score, which is positive and negative for TFs that are more active in asthma and no-asthma respectively. VIPER also produces the associated False Discovery Rate values (FDR_VIPER_), which were used in NeTFactor as a measure of the disease-related activity of the TFs constituting the GRN. This is also the goal of methods like MRA^16^ and its variants^17^.
3. Calculating the likelihood of a TF regulating the biomarker gene set: Even if a TF is determined to be active in a disease in the above step, it may not necessarily regulate the target biomarker, as it may be regulating other genes. Therefore, to determine the likelihood of a TF regulating the biomarker gene set, we used the Fisher’s exact test^32^ to assess the statistical significance of the number of genes in the overlapping the set of genes regulated by the TF in the GRN (its regulon) and the biomarker. We then used the Benjamini-Hochberg procedure^33^ to correct for multiple hypothesis testing and used the resulting FDR_BIOMARKER_ as the final likelihood of the TF regulating the biomarker gene set for further analysis.
4. Using convex optimization to find the most significant set of regulators: Since a gene in the biomarker may be regulated by multiple TFs, there may be redundancy (overlap) among the targets of the regulator TFs identified. To enhance NeTFactor’s ability to identify a set of regulators that reveal complementary aspects of disease biology, this step of the framework also takes into account the sets of biomarker genes each regulator targets. A possible *greedy* approach to this task is to incrementally select TFs by the number of biomarker genes they target in the corresponding GRN, until a certain fraction, say 80%, of all the biomarker genes has been covered. However, this approach does not tackle the redundancy issue, as several of the selected regulators may still regulate highly overlapping sets of biomarker genes. Therefore, NeTFactor adopts a convex optimization method for prioritizing candidate regulators based on the GRN inferred in Step 1 and the likelihoods calculated in Steps 2 and 3. Mathematically, this optimization problem can be formulated as

$$minimize\,\,||x|{|}_{0}\,s.t.\,{\rm{A}}x\ge 1,\,x\in \{0,1\},$$

where ${\rm{A}}$ is the (#TFs in GRN)X(#biomarker genes) matrix, with the *A*(*i*, *j*) entry representing the likelihood that TF *i* is active and regulating gene *j* in the biomarker set, which is defined as (1 − FDR_VIPER_) × (1 − FDR_BIOMARKER_) if *j* is a target of *i* in the GRN, and 0 otherwise.

The purpose of solving the above optimization problem stated is to find the value of the vector *x*, which contains one entry for each TF, such that there are as few non-zero entries in *x* as possible (purpose of the L_0_ norm) and each gene in the biomarker would be targeted/covered by at least one TF (denoted by the ${\rm{A}}x\,\ge 1$ condition) in the GRN. However, since this problem is known to be computationally intractable^68^, we relaxed it by using the L_1_ norm, thus changing the optimization problem to

$$minimize\,\,||x|{|}_{1}\,s.t.\,{\rm{A}}x\ge 1.$$

This problem is a derivative of the well-known LASSO algorithm, and thus can be solved using standard methods^69^ and packages like CVXR^70^. The decreasing magnitudes of the values constituting $x$ (LASSO weights) that are obtained by solving the above problem were used to rank the TFs in terms of their likelihood of non-redundantly regulating the biomarker.

Finally, to facilitate deeper examination of the top-ranking regulators, such as experimental validation in this study, we were most interested in determining the most significant subset of these regulator TFs that maximizes coverage of the biomarker. For this, we only selected TFs that were both significantly relevant to the disease (FDR_VIPER_ $\le \,$ 0.05) and the biomarker (FDR_BIOMARKER_ ≤ 0.05) for further study.

### Testing the robustness of NeTFactor’s results to the choice of the GRN inference algorithm

To test this aspect, we applied NeTFactor to the nasal RNAseq data by replacing the constituent ARACNE algorithm with GENIE3^29^, another well-established algorithm to infer GRNs. We used GENIE3’s publicly available R implementation (https://bioconductor.org/packages/release/bioc/html/GENIE3.html), setting the number of potential regulators of each gene, a required input parameter, to the default value of the square root of the total number of genes in the nasal RNAseq dataset. To compare the results obtained with the ARACNE-based ones, we selected the same number of top-weighted edges in the inferred GENIE3 network as the original nasal ARACNE GRN.

### Experimental validation

We used a human nasal epithelial primary cell line (PromoCell (Heidelberg, Germany)) at second passage for our experimental work, based on the rationale that a nasal epithelial cell line is from tissue closest in nature to the nasal brush samples that yielded the nasal RNAseq data used to develop the asthma biomarker. The cell line was grown according to manufacturer instructions in the presence of Primocin (Millipore Sigma, St Louis, MO, USA) at 1:500 dilution in Opti-MEM™ Reduced Serum Medium (Thermo Fisher Scientific, Waltham, MA, USA). Cells were cultured to a confluence of 70–90% in a 24-well plate. For pilot studies to determine optimal stimulant concentration, stimulations were done with lipopolysaccharide (LPS) (Millipore Sigma, St Louis, MO, USA) at 1 mg/mL, polyinosinic:polycytidylic acid (Poly (I:C)) (InvivoGen, San Diego, CA, USA) at 20 and 50 mg/mL, and immunostimulatory cytosine–phosphate–guanosine (CpG) (InvivoGen, San Diego, CA, USA) at 2.5 mM. Supernatants were collected at 6, 24, 48 and 72 hours and stored frozen at −80 °C for further experiments.

siRNA-mediated knockdown of PPARG and ETV4 mRNA was performed according to the protocol and supplies from ThermoFisher Scientific (RNAi Handbook, ThermoFisher Scientific, Waltham, MA, USA; thermofisher.com/RNAi). Cells were seeded at the sixth passage on a 24-well plate at ~2 × 10^4^ cell per well. At 48 hours, cell media were refreshed. siRNA for PPARG and ETV4, as well as the corresponding negative siRNA controls were each mixed with Lipofectamine RNAiMAX reagent and added to the well at 5 pmol/0.5 mL medium/well. At 72 hours following addition of the transfection mix, cells were stimulated with Poly(I:C) or media control for 24 hours. *Cytokine and chemokine* levels in supernatants were then measured using X Multiplex Human Cytokine/chemokine assay kits (Millipore Sigma, St. Louis, MO, USA).

### Testing the generalizability of NeTFactor to scenarios when the GRN and biomarker are derived from different data sets

To assess the generalizability of NetTFactor’s performance to instances when the GRN and biomarker are derived from different data sets, we also applied NeTFactor to a GRN derived from an independent dataset from a distinct cohort of children with asthma and controls^43^. This independent nasal transcriptome dataset was generated from 225 asthmatics and controls recruited separately from the original dataset. All subjects were recruited as part of an IRB-approved study at the Mount Sinai Health System, New York, NY and provided written informed consent.

Specifically, we constructed a new GRN using nasal transcriptome data from the independent cohort by applying ARACNE with 100 bootstraps and the seed set of 132 MSigDB TFs–the same settings used to derive the original asthma GRN. We then applied the same four steps of NetFactor as had been applied to the original GRN to this independently-derived GRN to identify potential regulators of the 90-gene asthma biomarker.

### Application of NeTFactor to a different disease biomarker

To assess NeTFactor’s ability to generalize to biomarkers of other diseases and/or phenotypes, we applied NeTFactor to identify TF regulators of a biomarker of peanut allergic reactions^44^. The data for GRN construction included whole blood transcriptome profiles from 40 peanut allergic children undergoing double-blind, placebo-controlled oral challenges to peanut. Longitudinal peripheral blood samples for whole blood transcriptome profiling were obtained during each challenge^44^. Here, peanut allergic reaction was the target phenotype, and the two classes were reaction (i.e. captured by samples obtained during peanut challenge), and no reaction (i.e. captured by samples obtained during placebo challenge)^44^. In the primary study of this cohort^44^, 26 key driver genes of peanut allergic reaction were identified (Supplementary Table 4 of this ref.^44^) and were considered the biomarker for NeTFactor.

We applied the NeTFactor framework to the gene expression data from the peanut allergy cohort and the biomarker of peanut allergic reactions^44^. For GRN inference using ARACNE, we used 100 bootstraps and the seed set of 221 MSigDB TFs, the same settings used to derive the original asthma GRN. The other steps of NeTFactor were applied exactly as in the original asthma case study, with the exception that VIPER was run with the gene-based permutation option to build the null hypothesis due to the fact that each subject had whole blood transcriptome profiles from longitudinal samples obtained during both peanut and placebo challenges, and samples were thus not independent within or between the classes.

### Ethics approval and consent to participate

The institutional review boards of Brigham & Women’s Hospital and the Icahn School of Medicine at Mount Sinai approved the study protocols. Written informed consent was obtained from all subjects and all research was performed in accordance with relevant guidelines and regulations.

## Software Availability

The code for NeTFactor implementation is available at https://github.com/GauravPandeyLab/NeTFactor.

## Supplementary information

Supplementary Materials

## Data Availability

RNAseq data from the asthma cohort that yielded the primary nasal gene expression data set used in this study are available at https://www.synapse.org/#!Synapse:syn9878922/files/ (10.7303/syn9878922). The RNAseq data for the peanut allergy cohort can be found at https://www.synapse.org/#!Synapse:syn10212437/files/ (10.7303/syn10212437).
