# Supplementary material for: NeTFactor, a framework for identifying transcriptional regulators of gene expression-based biomarkers
Source: Sci Rep. 2019 Sep 10;9:12970. doi: 10.1038/s41598-019-49498-y (PMC6737052; doi:10.1038/s41598-019-49498-y)
Supplement: Supplementary file 1 — Supplementary Materials [file 41598_2019_49498_MOESM1_ESM.docx]

**SUPPLEMENTARY MATERIALS**

**NeTFactor, a framework for identifying transcriptional regulators of gene expression-based biomarkers**

Mehmet Eren Ahsen^1^, Yoojin Chun^1^, Alexander Grishin^2^, Galina Grishina^2^, Gustavo Stolovitzky^1,3^, Gaurav Pandey^1,^* and Supinda Bunyavanich^1,2,^*

^1^ Icahn Institute for Genomics and Multiscale Biology and Department of Genetics and Genomic Sciences, Icahn School of Medicine at Mount Sinai, New York, NY, USA.

^2^ Division of Allergy & Immunology, Department of Pediatrics, Icahn School of Medicine

at Mount Sinai, New York, NY, USA.

^3^ IBM T.J. Watson Research Center, Yorktown Heights, New York, NY, USA

* Correspondence: [gaurav.pandey@mssm.edu](mailto:gaurav.pandey@mssm.edu) and [supinda.bunyavanich@mssm.edu](mailto:supinda.bunyavanich@mssm.edu)

**Supplementary Figure 1**: Percentage of genes in the asthma biomarker (*coverage*) regulated by increasing number of TFs selected by the greedy (red curve) and LASSO-based (blue curve) approaches in the final step of NeTFactor. The LASSO approach achieves higher coverage (#asthma biomarker genes covered) with fewer TFs than the greedy approach due to its better control of the redundancy among the sets of biomarker genes regulated by the selected TFs.

**Supplementary Table 1: Baseline characteristics of subjects in the RNAseq dataset**

|  | All  (n=150) | Asthma  (n=53) | No Asthma (n=97) |
| --- | --- | --- | --- |
| Age: years | 26.9 (5.4) | 25.7 (2.0) | 27.6 (6.5) |
| Sex: female | 89 (59.3%) | 24 (45.3%) | 65 (67.0%) |
| Race |  |  |  |
| Caucasian | 116 (77.3%) | 21 (40.4%) | 96 (99.0%) |
| African  American | 24 (16.0%) | 23 (43.4%) | 1 (1.0%) |
| Latino | 5 (3.3%) | 5 (9.4%) | 0 (0.0%) |
| Other | 5 (3.3%) | 4 (7.5%) | 0 (0.0%) |
| FEV1^A^:  % predicted | 94.7 (10.0) | 94.6% (10.9) | 94.8 (9.7) |
| FEV1/FVC^A^: % | 82.5 (6.4) | 81.5 (6.7) | 83.1 (6.3) |
| Bronchodilator response: % | 5.6 (6.0) | 8.7 (6.4) | 3.9 (5.1) |
| Age asthma onset: years |  | 3.2 (2.7) | n/a |
| Allergic rhinitis | 60 (40.0%) | 29 (54.7%) | 31 (32.0%) |
| Nasal steroids | 14 (9.3%) | 9 (17.0%) | 5 (5.2%) |
| Smoking | 7 (4.7%) | 1 (1.9%) | 6 (6.2%) |

Mean (SD) or Number (%) provided

^A^pre-bronchodilator measures. FEV1 = forced expiratory flow volume in 1 second, FVC = forced vital capacity

**Supplementary Table 2**: All TFs in the base nasal GRN (first column) in terms of LASSO weights (second column) produced by the final step of NeTFactor, indicating the TFs’ likelihood of regulating the asthma biomarker as significantly and exclusively as possible. The FDR values calculated in the two preceding steps of NeTFactor are also shown for reference, along with the number of biomarker genes regulated by each TF, as well as those cumulatively regulated by it and all the TFs preceding it.

| **TF** | **LASSO weight** | **FDR_VIPER_** | **FDR_BIOMARKER_** | **Number of biomarker**  **genes regulated** | **Cumulative number**  **of biomarker**  **genes regulated** |
| --- | --- | --- | --- | --- | --- |
| PPARG | 1.07218041 | 0.0151 | 0.00610428 | 16 | 16 |
| ETV4 | 1.01538838 | 0.0324 | 6.79E-05 | 15 | 24 |
| GTF2A2 | 1.01375917 | 0.0113 | 0.39510957 | 11 | 30 |
| EGR1 | 1.00650063 | 0.566 | 0.2856716 | 3 | 33 |
| SPI1 | 1.00288079 | 0.61 | 0.36337659 | 8 | 38 |
| CEBPB | 1.00076284 | 0.887 | 0.06204927 | 7 | 41 |
| XBP1 | 1.00049992 | 0.998 | 0.00020272 | 11 | 52 |
| SOX9 | 1 | 0.108 | 1 | 4 | 54 |
| BACH1 | 1 | 0.0502 | 1 | 5 | 57 |
| NF1 | 1 | 0.807 | 1 | 2 | 59 |
| FOXM1 | 1 | 0.983 | 1 | 4 | 62 |
| E4F1 | 1 | 0.108 | 1 | 1 | 63 |
| GABPB2 | 1 | 0.623 | 1 | 1 | 64 |
| ARNT | 1 | 0.965 | 1 | 1 | 65 |
| TCF12 | 1 | 0.946 | 1 | 1 | 66 |
| MAX | 1 | 0.564 | 1 | 2 | 67 |
| IRF1 | 1 | 0.759 | 1 | 2 | 68 |
| E2F1 | 1 | 0.983 | 1 | 2 | 69 |
| TFAP2A | 1 | 0.108 | 1 | 4 | 70 |
| TFDP1 | 1 | 0.896 | 1 | 2 | 71 |
| STAT1 | 1 | 0.807 | 1 | 2 | 73 |
| HSF1 | 0.71829337 | 0.0289 | 1 | 4 | 74 |
| PRRX2 | 0.59916635 | 0.998 | 1 | 5 | 75 |
| CDC5L | 0.53826315 | 0.443 | 1 | 2 | 76 |
| SREBF1 | 0.5 | 0.807 | 1 | 1 | 77 |
| E2F4 | 0.5 | 0.623 | 1 | 1 | 77 |
| AHR | 0.46173685 | 0.17 | 1 | 2 | 77 |
| STAT3 | 0.45042997 | 0.244 | 1 | 5 | 78 |
| TEF | 0.40083365 | 0.105 | 1 | 1 | 78 |
| GABPA | 0.28170663 | 0.105 | 1 | 1 | 78 |
| GTF2A1 | 0.14682918 | 0.781 | 1 | 2 | 78 |
| GTF3A | 0.13424695 | 0.623 | 1 | 1 | 78 |
| TEAD1 | 0.13424695 | 0.91 | 1 | 1 | 78 |
| RREB1 | 0.13424695 | 0.887 | 1 | 1 | 78 |
| NR2F2 | 0.03138564 | 0.196 | 0.278106 | 10 | 78 |
| ESR1 | 0.00990514 | 0.104 | 0.22837314 | 6 | 78 |
| PAX6 | 0.00748346 | 0.716 | 0.22837314 | 5 | 78 |
| MYB | 0.00738772 | 0.105 | 0.54717249 | 14 | 78 |
| PAX3 | 0.00713314 | 0.0113 | 0.38430822 | 4 | 78 |
| MEIS1 | 0.00265604 | 0.803 | 0.2856716 | 5 | 78 |
| ATF3 | 0.00068832 | 0.568 | 0.88323026 | 3 | 78 |
| CEBPD | 0.00038478 | 0.623 | 0.94497403 | 6 | 78 |
| TFDP2 | 5.10E-10 | 0.443 | 1 | 6 | 78 |
| NFE2L1 | 1.93E-10 | 0.0439 | 1 | 4 | 78 |
| MAF | 1.93E-10 | 0.105 | 1 | 4 | 78 |
| HIF1A | 1.93E-10 | 0.0289 | 1 | 2 | 78 |
| FOXJ1 | 1.93E-10 | 0.0151 | 1 | 14 | 78 |
| HLF | 1.93E-10 | 0.61 | 1 | 2 | 78 |
| PPARA | 1.93E-10 | 0.965 | 1 | 2 | 78 |
| TCF4 | 1.93E-10 | 0.769 | 1 | 3 | 78 |
| PBX1 | 1.93E-10 | 0.623 | 1 | 4 | 78 |
| GATA3 | 1.93E-10 | 0.769 | 1 | 1 | 78 |
| RELA | 1.93E-10 | 0.823 | 1 | 2 | 78 |
| DDIT3 | 1.93E-10 | 0.568 | 1 | 3 | 78 |
| ZFP161 | 1.93E-10 | 0.999 | 1 | 2 | 78 |
| SMAD3 | 1.93E-10 | 0.443 | 1 | 2 | 78 |
| ESRRA | 1.93E-10 | 0.58 | 1 | 2 | 78 |
| RFX1 | 1.93E-10 | 0.0175 | 1 | 2 | 78 |
| FOXA1 | 1.93E-10 | 0.623 | 1 | 2 | 78 |
| TFAP2C | 1.93E-10 | 0.108 | 1 | 3 | 78 |
| FOXQ1 | 1.93E-10 | 0.807 | 1 | 1 | 78 |
| MAFG | 1.93E-10 | 0.896 | 1 | 1 | 78 |
| ATF1 | 1.93E-10 | 0.105 | 1 | 2 | 78 |
| SRF | 1.93E-10 | 0.919 | 1 | 1 | 78 |
| LMO2 | 1.93E-10 | 0.715 | 1 | 1 | 78 |
| NRF1 | 1.93E-10 | 0.105 | 1 | 2 | 78 |
| ETS2 | 1.93E-10 | 0.207 | 1 | 1 | 78 |
| CBFA2T2 | 1.93E-10 | 0.568 | 1 | 1 | 78 |
| IRF7 | 1.93E-10 | 0.781 | 1 | 1 | 78 |
| CREB1 | 1.93E-10 | 0.141 | 1 | 1 | 78 |
| TFAP4 | 1.93E-10 | 0.105 | 1 | 1 | 78 |
| RUNX2 | 1.93E-10 | 0.105 | 1 | 1 | 78 |
| SP1 | 1.93E-10 | 0.105 | 1 | 1 | 78 |
| STAT5A | 1.93E-10 | 0.946 | 1 | 1 | 78 |
| UBP1 | 1.93E-10 | 0.807 | 1 | 1 | 78 |
| NFIL3 | 1.93E-10 | 0.243 | 1 | 1 | 78 |
| SF1 | 1.93E-10 | 0.566 | 1 | 1 | 78 |
| ELK1 | 1.93E-10 | 0.42 | 1 | 1 | 78 |
| GABPB1 | 1.93E-10 | 0.206 | 1 | 1 | 78 |
| RXRB | 1.93E-10 | 0.196 | 1 | 0 | 78 |
| RXRA | 1.93E-10 | 0.413 | 1 | 0 | 78 |
| ZNF238 | 1.93E-10 | 0.708 | 1 | 0 | 78 |
| JUN | 1.93E-10 | 0.802 | 1 | 0 | 78 |
| NR1H3 | 1.93E-10 | 0.85 | 1 | 0 | 78 |
| TFCP2 | 1.93E-10 | 0.983 | 1 | 0 | 78 |
| STAT6 | 1.93E-10 | 0.998 | 1 | 0 | 78 |
| ETV7 | 1.93E-10 | 0.998 | 1 | 0 | 78 |
| ATF2 | 1.93E-10 | 0.999 | 1 | 0 | 78 |
| GATA6 | 1.93E-10 | 0.965 | 1 | 0 | 78 |
| MTF1 | 1.93E-10 | 0.965 | 1 | 0 | 78 |
| SMAD4 | 1.93E-10 | 0.896 | 1 | 0 | 78 |
| POU2F1 | 1.93E-10 | 0.896 | 1 | 0 | 78 |
| ITGAL | 1.93E-10 | 0.807 | 1 | 0 | 78 |
| MEF2A | 1.93E-10 | 0.805 | 1 | 0 | 78 |
| STAT2 | 1.93E-10 | 0.781 | 1 | 0 | 78 |
| ATF4 | 1.93E-10 | 0.733 | 1 | 0 | 78 |
| ELF2 | 1.93E-10 | 0.707 | 1 | 0 | 78 |
| ETS1 | 1.93E-10 | 0.583 | 1 | 0 | 78 |
| CEBPG | 1.93E-10 | 0.568 | 1 | 0 | 78 |
| REL | 1.93E-10 | 0.566 | 1 | 0 | 78 |
| STAT5B | 1.93E-10 | 0.519 | 1 | 0 | 78 |
| MYC | 1.93E-10 | 0.465 | 1 | 0 | 78 |
| SMAD1 | 1.93E-10 | 0.443 | 1 | 0 | 78 |
| SP3 | 1.93E-10 | 0.443 | 1 | 0 | 78 |
| RB1 | 1.93E-10 | 0.413 | 1 | 0 | 78 |
| ZHX2 | 1.93E-10 | 0.402 | 1 | 0 | 78 |
| FOXJ2 | 1.93E-10 | 0.363 | 1 | 0 | 78 |
| NR3C1 | 1.93E-10 | 0.338 | 1 | 0 | 78 |
| ELF1 | 1.93E-10 | 0.243 | 1 | 0 | 78 |
| NFE2L2 | 1.93E-10 | 0.17 | 1 | 0 | 78 |
| ATF6 | 1.93E-10 | 0.105 | 1 | 0 | 78 |
| EGR3 | 1.93E-10 | 0.443 | 1 | 4 | 78 |
| HSF2 | 1.93E-10 | 0.277 | 1 | 7 | 78 |
| REST | 1.93E-10 | 0.443 | 1 | 1 | 78 |
| RORA | 1.93E-10 | 0.413 | 1 | 1 | 78 |
| REPIN1 | 1.93E-10 | 0.00142 | 1 | 7 | 78 |
| IRF8 | 1.93E-10 | 0.803 | 1 | 3 | 78 |
| MAZ | 1.93E-10 | 0.00142 | 1 | 5 | 78 |
| CEBPA | 1.93E-10 | 0.244 | 1 | 4 | 78 |
| DBP | 1.93E-10 | 0.443 | 1 | 2 | 78 |
| YY1 | 1.93E-10 | 0.0352 | 1 | 2 | 78 |
| ZNF384 | 1.93E-10 | 0.254 | 1 | 2 | 78 |
| PCBP1 | 1.93E-10 | 0.568 | 1 | 6 | 78 |
| RUNX1 | 1.32E-10 | 0.453 | 1 | 1 | 78 |
| IRF2 | 1.32E-10 | 0.803 | 1 | 1 | 78 |
| TP53 | 1.14E-10 | 0.333 | 1 | 4 | 78 |
| HMGA1 | 1.13E-10 | 0.42 | 1 | 11 | 78 |
| VDR | 1.13E-10 | 0.443 | 1 | 4 | 78 |
| FOXC1 | 1.13E-10 | 0.623 | 1 | 1 | 78 |
| TCF3 | 2.78E-11 | 0.568 | 1 | 2 | 78 |
| TBP | 1.70E-11 | 0.867 | 1 | 2 | 78 |

**Supplementary Table 3: Cytokine secretion by human nasal epithelial cells stimulated for 24 hours with Poly(I:C), LPS, and CpG.** Cytokine concentrations are presented as the mean of duplicates ± standard deviation in pg/mL.

| **Cytokine** | **Media** | **Poly(I:C)** | **LPS** | **CpG** |
| --- | --- | --- | --- | --- |
| IL6 | 954 ± 51 | 9523 ± 719 | 1349 ± 32 | 1248 ± 117 |
| IL8 | 561 ± 74 | 8951 ± 1749 | Yes^1^ | Yes^1^ |
| TGFβ | 54 | 35 | 49 | 73 |
| CCL2 | 26 ± 2 | 205 ± 78 | 40 ± 1 | 32 ±16 |
| TSLP | 18 ± 3 | 53 ± 42 | 68 ± 96 | 46 ± 10 |
| CCL17 | 4 ± 2 | 4 ± 6 | 4 ± 6 | 12 ± 5 |
| IL25 | n.d.* | n.d.* | n.d.* | n.d.* |
| IL33 | n.d.* | n.d. | 62 ± 87 | 80 ± 113 |

*Below detection limits.

^1^Significant upregulation of IL8 level in the culture supernatant was detected but the exact amount could not be calculated due to the technical difficulties with the assay.

**Supplementary Table 4**: Results from the application of NeTFactor to the asthma biomarker and a nasal GRN derived from an independent cohort. All the TFs in the GRN (first column) are ranked in terms of LASSO weights (second column) produced by the final step of NeTFactor, indicating the TFs’ likelihood of regulating the asthma biomarker as significantly and exclusively as possible. The FDR values calculated in the two preceding steps of NeTFactor are also shown for reference, along with the number of biomarker genes regulated by each TF, as well as those cumulatively regulated by it and all the TFs preceding it.

| **TF** | **LASSO weight** | **FDR_VIPER_** | **FDR_BIOMARKER_** | **Number of biomarker**  **genes regulated** | **Cumulative number**  **of biomarker**  **genes regulated** |
| --- | --- | --- | --- | --- | --- |
| GATA2 | 1.08245791 | 0.412 | 1.21E-05 | 12 | 12 |
| ETV4 | 1.00097899 | 0.997 | 0.02402619 | 8 | 20 |
| PPARG | 1.00088108 | 0.997 | 0.0087164 | 15 | 32 |
| ESR1 | 1.00078731 | 0.997 | 0.01132234 | 9 | 36 |
| JUN | 1.00052477 | 0.997 | 0.1890089 | 3 | 39 |
| DDIT3 | 1.0003816 | 0.997 | 0.0339444 | 7 | 45 |
| CEBPB | 1.00033283 | 0.997 | 0.01745909 | 7 | 48 |
| AR | 1.00031756 | 0.997 | 0.01131423 | 7 | 50 |
| ATF3 | 1.00026806 | 0.997 | 0.1890089 | 3 | 51 |
| XBP1 | 1.00010963 | 0.997 | 0.83760531 | 6 | 54 |
| SPI1 | 1.00005832 | 0.997 | 0.83760531 | 5 | 57 |
| LEF1 | 1.00005125 | 0.997 | 0.83760531 | 9 | 58 |
| GFI1 | 1.00004838 | 0.997 | 0.84565534 | 4 | 59 |
| NR2F2 | 1.00004398 | 0.997 | 0.86488511 | 4 | 61 |
| POU3F1 | 1.00000047 | 0.997 | 0.9983167 | 4 | 63 |
| SF1 | 1 | 0.997 | 1 | 3 | 66 |
| STAT1 | 1 | 0.997 | 1 | 1 | 67 |
| CREB1 | 1 | 0.997 | 1 | 3 | 68 |
| PPARA | 1 | 0.997 | 1 | 4 | 71 |
| NRF1 | 0.99862427 | 0.997 | 0.25960836 | 6 | 73 |
| SP1 | 0.53973216 | 0.997 | 1 | 3 | 74 |
| CEBPA | 0.50000038 | 0.997 | 1 | 2 | 76 |
| DBP | 0.50000038 | 0.997 | 1 | 2 | 78 |
| TEF | 0.49999962 | 0.997 | 1 | 2 | 78 |
| MIF | 0.49999962 | 0.997 | 1 | 2 | 78 |
| UBP1 | 0.46026784 | 0.997 | 1 | 2 | 78 |
| FOXC1 | 0.29262217 | 0.0779 | 0.15838894 | 5 | 78 |
| FOXI1 | 0.00199377 | 0.997 | 0.02402619 | 7 | 78 |
| LMO2 | 0.00191062 | 0.997 | 0.01132234 | 7 | 78 |
| MAF | 0.00096147 | 0.997 | 0.02402619 | 10 | 78 |
| EGR1 | 0.00058047 | 0.997 | 0.03354852 | 4 | 78 |
| NFE2 | 0.00056003 | 0.997 | 0.01131423 | 6 | 78 |
| BACH2 | 0.00024227 | 0.997 | 0.36117054 | 4 | 78 |
| PBX1 | 6.05E-05 | 0.997 | 0.83760531 | 4 | 78 |
| POU6F1 | 1.97E-05 | 0.997 | 0.94060721 | 4 | 78 |
| E2F1 | 5.58E-09 | 0.997 | 1 | 2 | 78 |
| RREB1 | 3.72E-09 | 0.997 | 1 | 3 | 78 |
| STAT2 | 1.72E-09 | 0.997 | 1 | 2 | 78 |
| ARNT | 1.48E-09 | 0.997 | 1 | 2 | 78 |
| GTF2A2 | 1.48E-09 | 0.997 | 1 | 3 | 78 |
| HSF2 | 1.48E-09 | 0.997 | 1 | 2 | 78 |
| CBFA2T2 | 1.48E-09 | 0.997 | 1 | 5 | 78 |
| RUNX2 | 1.14E-09 | 0.997 | 1 | 3 | 78 |
| GABPB2 | 1.14E-09 | 0.997 | 1 | 2 | 78 |
| TFAP2A | 1.14E-09 | 0.997 | 1 | 2 | 78 |
| IRF7 | 1.14E-09 | 0.997 | 1 | 2 | 78 |
| ETS2 | 1.14E-09 | 0.997 | 1 | 4 | 78 |
| EGR3 | 1.14E-09 | 0.997 | 1 | 2 | 78 |
| MAFG | 1.14E-09 | 0.997 | 1 | 1 | 78 |
| PAX3 | 1.14E-09 | 0.997 | 1 | 1 | 78 |
| REST | 1.14E-09 | 0.997 | 1 | 1 | 78 |
| NFIL3 | 1.14E-09 | 0.997 | 1 | 1 | 78 |
| STAT5B | 1.14E-09 | 0.997 | 1 | 1 | 78 |
| BACH1 | 1.14E-09 | 0.997 | 1 | 1 | 78 |
| FOXQ1 | 1.14E-09 | 0.997 | 1 | 2 | 78 |
| TFAP2C | 1.14E-09 | 0.997 | 1 | 3 | 78 |
| YY1 | 1.14E-09 | 0.997 | 1 | 1 | 78 |
| EGR2 | 1.14E-09 | 0.997 | 1 | 0 | 78 |
| PAX8 | 1.14E-09 | 0.997 | 1 | 0 | 78 |
| STAT4 | 1.14E-09 | 0.997 | 1 | 0 | 78 |
| IRF8 | 1.14E-09 | 0.997 | 1 | 0 | 78 |
| ITGAL | 1.14E-09 | 0.997 | 1 | 0 | 78 |
| CBFA2T3 | 1.14E-09 | 0.997 | 1 | 0 | 78 |
| STAT5A | 1.14E-09 | 0.997 | 1 | 0 | 78 |
| TFCP2 | 1.14E-09 | 0.997 | 1 | 0 | 78 |
| RXRB | 1.14E-09 | 0.997 | 1 | 0 | 78 |
| STAT6 | 1.14E-09 | 0.997 | 1 | 0 | 78 |
| E2F4 | 1.14E-09 | 0.997 | 1 | 0 | 78 |
| TFAP4 | 1.14E-09 | 0.997 | 1 | 0 | 78 |
| GTF2A1 | 1.14E-09 | 0.997 | 1 | 0 | 78 |
| ELF1 | 1.14E-09 | 0.997 | 1 | 0 | 78 |
| ATF2 | 1.14E-09 | 0.997 | 1 | 0 | 78 |
| HIF1A | 1.14E-09 | 0.997 | 1 | 0 | 78 |
| NR6A1 | 1.14E-09 | 0.997 | 1 | 0 | 78 |
| RXRA | 1.14E-09 | 0.997 | 1 | 0 | 78 |
| TBP | 1.14E-09 | 0.997 | 1 | 0 | 78 |
| SMAD4 | 1.14E-09 | 0.997 | 1 | 0 | 78 |
| SOX9 | 1.14E-09 | 0.997 | 1 | 0 | 78 |
| ZNF384 | 1.14E-09 | 0.997 | 1 | 0 | 78 |
| NR3C1 | 1.14E-09 | 0.997 | 1 | 0 | 78 |
| REPIN1 | 1.14E-09 | 0.997 | 1 | 0 | 78 |
| FOXJ2 | 1.14E-09 | 0.997 | 1 | 0 | 78 |
| RB1 | 1.14E-09 | 0.997 | 1 | 0 | 78 |
| SREBF1 | 1.14E-09 | 0.997 | 1 | 0 | 78 |
| TEAD1 | 1.14E-09 | 0.997 | 1 | 0 | 78 |
| NF1 | 1.14E-09 | 0.997 | 1 | 0 | 78 |
| FOXN1 | 1.14E-09 | 0.997 | 1 | 0 | 78 |
| GABPB1 | 1.14E-09 | 0.997 | 1 | 0 | 78 |
| MEF2A | 1.14E-09 | 0.997 | 1 | 0 | 78 |
| SOX5 | 1.14E-09 | 0.997 | 1 | 0 | 78 |
| CEBPG | 1.14E-09 | 0.997 | 1 | 0 | 78 |
| PAX6 | 1.14E-09 | 0.997 | 1 | 0 | 78 |
| NKX3-1 | 1.14E-09 | 0.997 | 1 | 0 | 78 |
| GATA6 | 1.14E-09 | 0.997 | 1 | 3 | 78 |
| MAX | 1.14E-09 | 0.997 | 1 | 1 | 78 |
| GABPA | 1.14E-09 | 0.997 | 1 | 1 | 78 |
| IRF2 | 1.14E-09 | 0.997 | 1 | 1 | 78 |
| MTF1 | 1.14E-09 | 0.997 | 1 | 1 | 78 |
| GATA3 | 1.14E-09 | 0.997 | 1 | 1 | 78 |
| REL | 1.14E-09 | 0.997 | 1 | 2 | 78 |
| ETS1 | 1.14E-09 | 0.997 | 1 | 2 | 78 |
| POU2F1 | 1.14E-09 | 0.997 | 1 | 1 | 78 |
| SMAD1 | 1.14E-09 | 0.997 | 1 | 1 | 78 |
| NFE2L1 | 1.14E-09 | 0.997 | 1 | 2 | 78 |
| ATF4 | 1.14E-09 | 0.997 | 1 | 4 | 78 |
| ZHX2 | 1.14E-09 | 0.997 | 1 | 1 | 78 |
| MYC | 1.14E-09 | 0.997 | 1 | 1 | 78 |
| SP3 | 1.14E-09 | 0.997 | 1 | 2 | 78 |
| TCF12 | 1.14E-09 | 0.997 | 1 | 2 | 78 |
| HLF | 1.14E-09 | 0.997 | 1 | 2 | 78 |
| CDC5L | 1.14E-09 | 0.997 | 1 | 1 | 78 |
| TFDP1 | 1.14E-09 | 0.997 | 1 | 1 | 78 |
| TCF4 | 1.14E-09 | 0.997 | 1 | 1 | 78 |
| RELA | 1.14E-09 | 0.997 | 1 | 1 | 78 |
| TFDP2 | 1.14E-09 | 0.997 | 1 | 1 | 78 |
| HSF1 | 1.14E-09 | 0.997 | 1 | 1 | 78 |
| MEIS1 | 1.14E-09 | 0.997 | 1 | 3 | 78 |
| RFX1 | 1.14E-09 | 0.997 | 1 | 2 | 78 |
| AHR | 1.14E-09 | 0.997 | 1 | 3 | 78 |
| VDR | 1.14E-09 | 0.997 | 1 | 5 | 78 |
| FOXJ1 | 1.14E-09 | 0.997 | 1 | 9 | 78 |
| NFE2L2 | 1.14E-09 | 0.997 | 1 | 3 | 78 |
| PCBP1 | 1.14E-09 | 0.997 | 1 | 3 | 78 |
| ETV7 | 1.14E-09 | 0.997 | 1 | 2 | 78 |
| IRF1 | 1.14E-09 | 0.997 | 1 | 1 | 78 |
| PRRX2 | 1.14E-09 | 0.997 | 1 | 1 | 78 |
| FOXA1 | 1.14E-09 | 0.997 | 1 | 3 | 78 |
| HMGA1 | 1.14E-09 | 0.997 | 1 | 1 | 78 |
| ATF1 | 1.14E-09 | 0.997 | 1 | 2 | 78 |
| KLF12 | 1.14E-09 | 0.997 | 1 | 2 | 78 |
| ATF6 | 1.14E-09 | 0.997 | 1 | 3 | 78 |
| SRF | 1.14E-09 | 0.997 | 1 | 3 | 78 |
| STAT3 | 1.12E-09 | 0.997 | 1 | 1 | 78 |
| RORA | 1.12E-09 | 0.997 | 1 | 4 | 78 |
| E4F1 | 8.32E-10 | 0.997 | 1 | 1 | 78 |
| NR1H3 | 8.32E-10 | 0.997 | 1 | 1 | 78 |
| ELK1 | 8.32E-10 | 0.997 | 1 | 1 | 78 |
| TCF3 | 8.32E-10 | 0.997 | 1 | 2 | 78 |
| TP53 | 8.31E-10 | 0.997 | 1 | 2 | 78 |
| CEBPD | 8.31E-10 | 0.997 | 1 | 2 | 78 |
| ELF2 | 8.25E-10 | 0.997 | 1 | 3 | 78 |
| RUNX1 | 7.91E-10 | 0.997 | 1 | 1 | 78 |
| GTF3A | 7.91E-10 | 0.997 | 1 | 1 | 78 |
| FOXM1 | 6.75E-10 | 0.997 | 1 | 3 | 78 |
| SMAD3 | 6.14E-10 | 0.997 | 1 | 4 | 78 |
| MYB | 6.13E-10 | 0.997 | 1 | 9 | 78 |
| MAZ | 6.12E-10 | 0.997 | 1 | 1 | 78 |
| ESRRA | 6.12E-10 | 0.997 | 1 | 1 | 78 |

**Supplementary Table 5**: Results from the application of NeTFactor to a peanut allergy biomarker and relevant GRN. All the TFs in the GRN (first column) are ranked in terms of LASSO weights (second column) produced by the final step of NeTFactor, indicating the TFs’ likelihood of regulating the biomarker as significantly and exclusively as possible. The FDR values calculated in the two preceding steps of NeTFactor are also shown for reference, along with the number of biomarker genes regulated by each TF, as well as those cumulatively regulated by it and all the TFs preceding it.

| **TF** | **LASSO weight** | **FDR_VIPER_** | **FDR_BIOMARKER_** | **Number of biomarker**  **genes regulated** | **Cumulative number**  **of biomarker**  **genes regulated** |
| --- | --- | --- | --- | --- | --- |
| RORA | 1.01899104 | 2.98E-08 | 0.3550764 | 3 | 3 |
| RXRA | 1.01866107 | 5.56E-20 | 0.54285478 | 7 | 9 |
| NFIL3 | 0.95087087 | 1.12E-22 | 0.0083496 | 6 | 13 |
| SPI1 | 0.91432946 | 2.44E-08 | 0.01037404 | 7 | 17 |
| STAT3 | 0.69800646 | 1.41E-05 | 3.34E-05 | 8 | 19 |
| STAT6 | 0.5729584 | 0.0122 | 0.00553213 | 6 | 21 |
| VDR | 0.51546284 | 0.00398 | 0.01141138 | 4 | 21 |
| ELF1 | 0.33333333 | 7.67E-74 | 1 | 1 | 22 |
| CDC5L | 0.33333333 | 2.41E-32 | 1 | 1 | 22 |
| TCF3 | 0.33333333 | 3.31E-21 | 1 | 1 | 22 |
| ELF2 | 0.18437504 | 4.80E-20 | 0.12301435 | 5 | 22 |
| NR6A1 | 0.18013254 | 1.11E-51 | 1 | 2 | 23 |
| GTF2A1 | 0.16397349 | 0 | 1 | 1 | 23 |
| REL | 0.16397349 | 9.88E-228 | 1 | 1 | 23 |
| TBP | 0.16397349 | 1.32E-212 | 1 | 1 | 23 |
| MIF | 0.16397349 | 1.85E-201 | 1 | 1 | 23 |
| MAZ | 0.16397349 | 7.75E-127 | 1 | 1 | 23 |
| ETS2 | 0.12412523 | 0.0118 | 0.00553213 | 5 | 23 |
| IRF2 | 0.1237878 | 0.0633 | 0.26417409 | 3 | 23 |
| IRF1 | 0.07002045 | 1.50E-22 | 0.025938 | 5 | 23 |
| CEBPB | 0.06695705 | 9.44E-30 | 0.05385878 | 5 | 23 |
| HIF1A | 0.05010551 | 0.115 | 0.31309472 | 2 | 23 |
| SMAD3 | 0.04387911 | 0.0885 | 0.04539778 | 4 | 23 |
| BACH1 | 0.02704871 | 3.25E-53 | 0.29990016 | 4 | 23 |
| NFE2 | 0.02408172 | 0.00097 | 0.23012374 | 3 | 23 |
| LEF1 | 0.01011128 | 0.385 | 0.29990016 | 3 | 23 |
| GCM1 | 0.00869706 | 0.391 | 0.29990016 | 2 | 23 |
| BACH2 | 0.00439041 | 0.133 | 0.74278668 | 2 | 23 |
| STAT5B | 0.00353805 | 0.804 | 0.00553213 | 4 | 23 |
| CEBPD | 0.00243442 | 2.85E-38 | 0.86160687 | 3 | 23 |
| DDIT3 | 0.00025408 | 0.983 | 0.15660191 | 3 | 23 |
| E2F4 | 1.77E-10 | 3.35E-28 | 1 | 2 | 23 |
| ATF6 | 1.77E-10 | 2.99E-42 | 1 | 3 | 23 |
| SP3 | 1.77E-10 | 1.54E-13 | 1 | 1 | 23 |
| SOX5 | 1.77E-10 | 0.34 | 1 | 1 | 23 |
| ESRRA | 1.77E-10 | 2.09E-51 | 1 | 1 | 23 |
| FOXJ2 | 1.77E-10 | 1 | 0.74278668 | 2 | 23 |
| ZNF384 | 1.77E-10 | 5.68E-12 | 1 | 1 | 23 |
| TFDP1 | 1.77E-10 | 2.72E-06 | 1 | 1 | 23 |
| MTF1 | 1.77E-10 | 2.93E-22 | 1 | 1 | 23 |
| NR3C1 | 1.77E-10 | 8.45E-32 | 1 | 1 | 23 |
| NFE2L2 | 1.77E-10 | 6.15E-13 | 1 | 1 | 23 |
| RUNX2 | 1.77E-10 | 1.37E-44 | 1 | 1 | 23 |
| PCBP1 | 1.77E-10 | 5.71E-09 | 1 | 1 | 23 |
| HSF2 | 1.77E-10 | 6.46E-19 | 1 | 1 | 23 |
| SMAD4 | 1.77E-10 | 6.81E-14 | 1 | 1 | 23 |
| SRF | 1.77E-10 | 2.89E-62 | 1 | 1 | 23 |
| CREB1 | 1.77E-10 | 8.92E-67 | 1 | 0 | 23 |
| ARNT | 1.77E-10 | 7.75E-63 | 1 | 0 | 23 |
| NF1 | 1.77E-10 | 7.14E-54 | 1 | 0 | 23 |
| MEF2A | 1.77E-10 | 2.78E-44 | 1 | 0 | 23 |
| ETV4 | 1.77E-10 | 6.35E-44 | 1 | 0 | 23 |
| AR | 1.77E-10 | 4.24E-39 | 1 | 0 | 23 |
| RB1 | 1.77E-10 | 1.15E-36 | 1 | 0 | 23 |
| ATF2 | 1.77E-10 | 8.21E-36 | 1 | 0 | 23 |
| SMAD1 | 1.77E-10 | 3.98E-33 | 1 | 0 | 23 |
| FOXM1 | 1.77E-10 | 1.36E-31 | 1 | 0 | 23 |
| MYB | 1.77E-10 | 9.04E-23 | 1 | 0 | 23 |
| NR1H3 | 1.77E-10 | 1.46E-18 | 1 | 0 | 23 |
| REST | 1.77E-10 | 1.21E-16 | 1 | 0 | 23 |
| TFCP2 | 1.77E-10 | 1.44E-15 | 1 | 0 | 23 |
| POU2F1 | 1.77E-10 | 8.43E-10 | 1 | 0 | 23 |
| TFDP2 | 1.77E-10 | 3.38E-09 | 1 | 0 | 23 |
| PAX8 | 1.77E-10 | 3.14E-08 | 1 | 0 | 23 |
| GABPB2 | 1.77E-10 | 8.87E-07 | 1 | 0 | 23 |
| STAT4 | 1.77E-10 | 9.49E-07 | 1 | 0 | 23 |
| PAX5 | 1.77E-10 | 0.00661 | 1 | 0 | 23 |
| MEIS1 | 1.77E-10 | 0.0545 | 1 | 0 | 23 |
| RUNX1 | 1.77E-10 | 0.246 | 1 | 0 | 23 |
| TCF4 | 1.77E-10 | 0.501 | 1 | 0 | 23 |
| STAT2 | 1.77E-10 | 0.781 | 1 | 0 | 23 |
| NKX3-1 | 1.77E-10 | 1 | 1 | 0 | 23 |
| ATF3 | 1.77E-10 | 1 | 1 | 0 | 23 |
| ESR1 | 1.77E-10 | 1 | 1 | 0 | 23 |
| HLF | 1.77E-10 | 1 | 1 | 0 | 23 |
| GTF2A2 | 1.77E-10 | 0.991 | 1 | 0 | 23 |
| ETV7 | 1.77E-10 | 0.966 | 1 | 0 | 23 |
| POU6F1 | 1.77E-10 | 0.775 | 1 | 0 | 23 |
| AHR | 1.77E-10 | 0.62 | 1 | 0 | 23 |
| STAT1 | 1.77E-10 | 0.613 | 1 | 0 | 23 |
| UBP1 | 1.77E-10 | 0.335 | 1 | 0 | 23 |
| CEBPG | 1.77E-10 | 0.309 | 1 | 0 | 23 |
| PPARA | 1.77E-10 | 0.258 | 1 | 0 | 23 |
| TAL1 | 1.77E-10 | 0.253 | 1 | 0 | 23 |
| ATF1 | 1.77E-10 | 0.165 | 1 | 0 | 23 |
| MAF | 1.77E-10 | 0.108 | 1 | 0 | 23 |
| YY1 | 1.77E-10 | 0.077 | 1 | 0 | 23 |
| RXRB | 1.77E-10 | 0.0354 | 1 | 0 | 23 |
| E2F1 | 1.77E-10 | 0.0313 | 1 | 0 | 23 |
| GATA2 | 1.77E-10 | 0.0139 | 1 | 0 | 23 |
| XBP1 | 1.77E-10 | 0.00135 | 1 | 0 | 23 |
| GFI1 | 1.77E-10 | 0.000749 | 1 | 0 | 23 |
| ZHX2 | 1.77E-10 | 0.000395 | 1 | 0 | 23 |
| GATA1 | 1.77E-10 | 0.000302 | 1 | 0 | 23 |
| ITGAL | 1.77E-10 | 0.000242 | 1 | 0 | 23 |
| ETS1 | 1.77E-10 | 0.000187 | 1 | 0 | 23 |
| MAFG | 1.77E-10 | 4.81E-05 | 1 | 0 | 23 |
| RREB1 | 1.77E-10 | 4.33E-05 | 1 | 0 | 23 |
| CBFA2T3 | 1.77E-10 | 2.90E-05 | 1 | 0 | 23 |
| IRF7 | 1.77E-10 | 3.23E-07 | 1 | 0 | 23 |
| SF1 | 1.77E-10 | 1.72E-07 | 1 | 0 | 23 |
| MYC | 1.77E-10 | 6.73E-08 | 1 | 0 | 23 |
| RFX1 | 1.77E-10 | 1.14E-08 | 1 | 0 | 23 |
| HSF1 | 1.77E-10 | 5.27E-12 | 1 | 0 | 23 |
| NFE2L1 | 1.77E-10 | 1.73E-12 | 1 | 0 | 23 |
| RELA | 1.77E-10 | 1.21E-16 | 1 | 0 | 23 |
| TEF | 1.77E-10 | 9.81E-17 | 1 | 0 | 23 |
| SREBF1 | 1.77E-10 | 1.75E-20 | 1 | 0 | 23 |
| MAX | 1.77E-10 | 2.13E-25 | 1 | 0 | 23 |
| FOXJ1 | 1.77E-10 | 1.20E-25 | 1 | 0 | 23 |
| E4F1 | 1.77E-10 | 1.40E-41 | 1 | 0 | 23 |
| NRF1 | 1.77E-10 | 6.66E-56 | 1 | 0 | 23 |
| ATF4 | 1.77E-10 | 8.08E-63 | 1 | 0 | 23 |
| JUN | 1.77E-10 | 6.77E-66 | 1 | 0 | 23 |
| CEBPA | 1.77E-10 | 1.17E-66 | 1 | 0 | 23 |
| IRF8 | 1.77E-10 | 3.26E-70 | 1 | 0 | 23 |
| REPIN1 | 1.77E-10 | 9.15E-74 | 1 | 0 | 23 |
| ELK1 | 1.77E-10 | 7.05E-84 | 1 | 0 | 23 |
| TFAP4 | 1.77E-10 | 2.22E-93 | 1 | 0 | 23 |
| CRX | 1.77E-10 | 5.04E-105 | 1 | 0 | 23 |
| DBP | 1.77E-10 | 1.26E-108 | 1 | 0 | 23 |
| GABPB1 | 1.77E-10 | 1.21E-10 | 1 | 2 | 23 |
| SP1 | 1.77E-10 | 4.29E-14 | 1 | 1 | 23 |
| LMO2 | 1.77E-10 | 0.000116 | 1 | 1 | 23 |
| KLF12 | 1.77E-10 | 5.54E-15 | 1 | 2 | 23 |
| GATA3 | 1.77E-10 | 0.0885 | 1 | 1 | 23 |
| TP53 | 1.77E-10 | 0.00514 | 1 | 1 | 23 |
| CBFA2T2 | 1.77E-10 | 6.61E-22 | 1 | 1 | 23 |
| HMGA1 | 1.77E-10 | 1.53E-130 | 1 | 1 | 23 |
| GABPA | 1.77E-10 | 2.64E-21 | 1 | 1 | 23 |
| PAX6 | 1.77E-10 | 0.0634 | 1 | 1 | 23 |
| STAT5A | 1.77E-10 | 1.33E-12 | 1 | 1 | 23 |
| PBX1 | 1.77E-10 | 0.0885 | 1 | 1 | 23 |
| TCF12 | 1.77E-10 | 5.74E-11 | 1 | 1 | 23 |
| GTF3A | 1.77E-10 | 1.61E-39 | 1 | 1 | 23 |
